# PFAS-Induced Charge Regulation and Aggregation in Polystyrene Nanoplastic Colloids

**DOI:** 10.1021/acs.jpclett.6c00866

**Published:** 2026-04-30

**Authors:** Tamás Péter, Dóra Takács, Bojana Katana, Şeyma Miray Simav, Gergő Terjéki, Viktória Hornok, Szilárd Sáringer, Matija Tomšič, István Szilágyi

**Affiliations:** † MTA-SZTE Momentum Biocolloids Research Group, Department of Physical Chemistry and Materials Science, Interdisciplinary Centre of Excellence, 37442 University of Szeged, H-6720 Szeged, Hungary; ‡ Institute of Condensed Matter and Nanosciences - Bio and Soft Matter, 83415 Université Catholique de Louvain, B-1348 Louvain-la-Neuve, Belgium; § Faculty of Chemistry and Chemical Technology, 37663 University of Ljubljana, Večna pot 113, SI-1000 Ljubljana, Slovenia

## Abstract

The co-occurrence of the smallest fraction of plastic debris (nanoplastics, NPLs) together with perfluoroalkyl and polyfluoroalkyl substances (PFASs) represents a major emerging environmental and health risk. Although their combined presence in aquatic systems is increasingly recognized, the physicochemical mechanisms governing their colloidal and interfacial behavior remain poorly understood. Here, the particle charge and aggregation mechanism of polystyrene NPLs were comprehensively studied in the presence of C8 PFASs differing in head groups. In oppositely charged systems, PFAS adsorption on NPLs leads to charge neutralization and reversal at appropriate mass ratios. Rapid particle aggregation was observed near the isoelectric points, where electrical double layers were absent. In contrast, at low and high PFAS concentrations, the development of substantial particle charge gives rise to electrostatically stabilized colloids. The dispersion stability was sensitive to the ionic strength at partial PFAS coverage of the NPL surface, while it was insensitive at high extent of PFAS adsorption. The results provide a unique physicochemical insight into the interfacial and aggregation processes relevant in aqueous systems containing both NPLs and PFASs in various environmental compartments.

## Graphical abstract

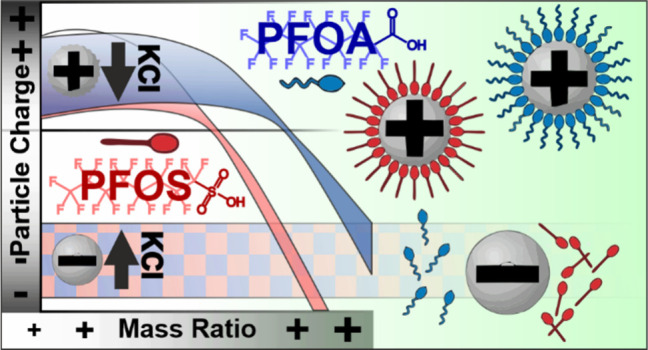

Nanoplastics (NPLs), typically defined as plastic particles with submicron dimensions, are now recognized as ubiquitous contaminants in aquatic and terrestrial environments.^1–3^ Their small size, high surface-to-volume ratio, and persistent polymeric nature enable long residence times, difficult remediation, and efficient transport as well as extensive interfacial interactions with dissolved species.^4–6^ Increasing evidence suggests that NPLs can interact with biological systems at the cellular and molecular levels, raising concerns about their potential ecological and human health impacts^7–10^ and highlighting the need of a global evidence-based regulatory framework that is currently under development.^11^

In parallel, per- and polyfluoroalkyl substances (PFASs, forever chemicals) constitute a large class of synthetic amphiphilic compounds characterized by extraordinary chemical stability and high mobility in water.^12–15^ Their widespread environmental occurrence, persistence, and bioaccumulation potential have made PFASs a focal point of current environmental research.^16,17^ The co-occurrence of NPLs and PFASs in aquatic systems is therefore increasingly recognized as an emerging issue of combined concern.^18^

Recent experimental and computational studies have demonstrated that PFASs readily adsorb on particles^19^ including NPLs and larger plastic grains via a combination of hydrophobic interactions, electrostatics, and headgroup specific effects.^20–23^ Molecular-level insight into PFAS adsorption on polystyrene NPLs has been provided by combined experimental-simulation approaches, revealing preferred adsorption geometries and the role of surface charge in determining adsorption strength.^24,25^ These interactions may influence PFAS bioavailability and toxicity, for example, through protein corona formation or altered cellular uptake pathways.^26–28^

Despite these advances, most existing studies emphasize adsorption capacity, sorption mechanisms, or toxicological end points, while the colloid chemistry consequences of PFAS adsorption remain insufficiently explored. In particular, how PFAS adsorption modifies NPL surface charge, interparticle forces, and aggregation kinetics is still poorly understood, even though these properties directly control dispersion stability, accumulation, and environmental transport.^18^ From the physical chemistry perspective, NPL stability in aqueous media may be governed by the balance between electrostatic repulsion, van der Waals attraction, and contributions of additional interparticle forces,^29^ all of which may be perturbed by the adsorption of amphiphilic PFAS molecules. The PFAS adsorption is expected to induce charge regulation effects including partial charge screening, neutralization, or even reversal, depending on surface coverage, ionic strength, and PFAS headgroup chemistry.^30^ Such effects are well-known to dramatically influence aggregation behavior in other colloidal systems, yet systematic experimental evidence directly linking PFAS-induced charge regulation to NPL aggregation is scarce.

Here, a systematic colloid and interface chemistry investigation of PFAS-induced charging features and particle aggregation in polystyrene NPL dispersions is presented. Positively (NPL­(+)) and negatively (NPL(−)) charged particles were studied in the presence of two representative C8 PFASs, perfluorooctanoic acid (PFOA) and perfluorooctanesulfonate (PFOS) (see Table S1), using light scattering (electrophoretic (ELS) and dynamic (DLS)) techniques, which have proven to be excellent tools to study NPL colloids.^31,32^

The hydrodynamic radii of the spherical NPL particles obtained by DLS were 53.2 nm for NPL­(+) and 66.6 nm for NPL(−), while transmission electron microscopy (TEM) imaging (see Figure S1) yielded 40.4 nm for NPL­(+) and 44.6 nm for NPL(−), respectively (Figure S2). The difference originates from the finite polydispersity of the samples (see PDI values in Table S2), which shifts the size toward larger values in light scattering experiments. As reflected by the pH-dependent electrophoretic mobility (EM) data measured in aqueous dispersions (Figure S3), surface functionalization led to significant positive and negative charge accumulation for NPL­(+) and NPL(−), respectively. Further measurements were carried out at pH 4 to check the effect of the sign of the NPL charge on the interaction with PFAS compounds, as discussed later.

Salinity is an important parameter in aqueous environments; various dissolved electrolytes of different concentrations and valence may contribute to the overall ionic strength. Therefore, the charge and aggregation data were assessed at different dissolved salt levels and expressed as EM and stability ratio shown in Figure 1a,b, respectively.

**Figure 1:**
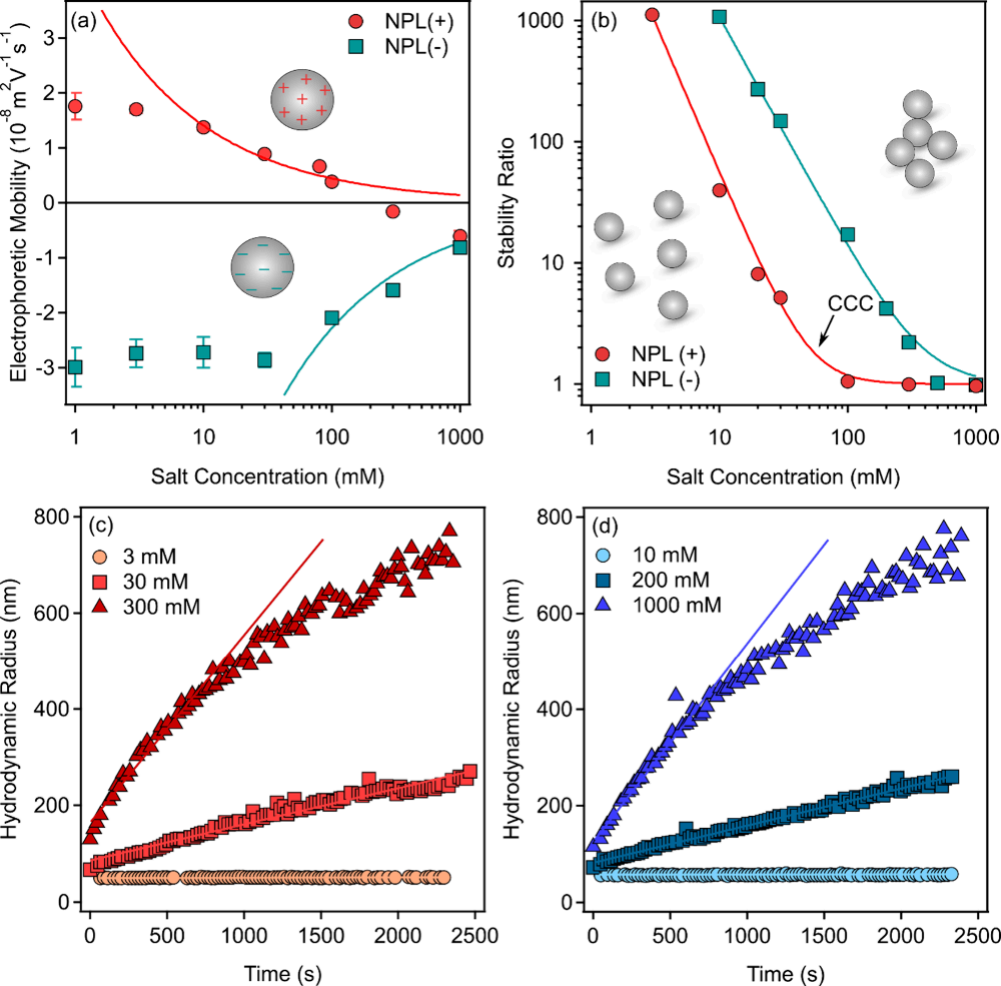
(a) Electrophoretic mobility and (b) stability ratio data as a function of salt concentration adjusted with KCl. The particle concentration was 10 mg/L. The solid lines in (a) indicate the data calculated with the Debye–Hückel model using eq S2, while in (b), they are the results of fits on the experimental data using eq S5. The time-dependent hydrodynamic radii for (c) NPL­(+) and (d) NPL(−) illustrate the differences in aggregation rates by varying the salt concentration. These data were used to calculate the aggregation rate constants using eq S3 and, subsequently, stability ratios with eq S4.

The magnitude of the EM decreased with an increase in the salt concentration at both NPLs and remained close to zero at high salinity. This is due to the classical salt screening effect leading to the shrinking of the electrical double layer (EDL) around the charged particles.^33^ The mobilities were converted to zeta potentials (eq S1) to determine the particle charge at the slip plane using the Debye–Hückel model (eq S2).^34^ The calculated data were in reasonable agreement with the experimental ones only at higher ionic strengths, because at lower salt concentrations the electrophoretic mobility is affected by electrokinetic contributions, such as retardation forces due to the extended electrical double layers, leading to deviations from the Debye–Hückel model predictions.^35^ The charge densities determined in this region indicated a five times higher absolute value for the NPL(−), most likely due to the larger density of the surface functional groups.

The NPL aggregation was investigated in time-resolved DLS experiments at various salt levels. The rate of aggregation for both NPL­(+) (Figure 1c) and NPL(−) (Figure 1d) was sensitive to the ionic strength indicated by different extents of increase in the radii versus time data. In general, stable NPL dispersions, i.e., no aggregation, were observed at low KCl concentrations, while the aggregation rates increased at an elevated concentration of salt.

The stability ratios were calculated from the kinetic data (eq S4) and plotted against the salt concentration. The trend for both NPL­(+) and NPL(−) was very similar; i.e., the values decreased with the ionic strength and reached unity at the critical coagulation concentration (CCC) calculated by fitting the experimental data with eq S5. Note that a stability ratio of one means that all particle collisions result in dimer formation, and hence, the aggregation is controlled solely by the diffusion of the NPLs in the medium. When the CCC data was compared (Table S2), the one determined for NPL(−) was about 5-times larger compared to the NPL­(+) case. This relation is in agreement with the charge density data discussed previously and implies electrostatic stabilization of the NPLs. Accordingly, the overall interparticle force is equal to the sum of EDL repulsion and van der Waals attraction.^29,30,34^ This was further confirmed by comparing the experimentally determined CCCs to the data calculated from the EM values using a recently developed model^36^ assuming the presence of the above-mentioned interactions. The CCCs were 56 and 398 mM for NPL­(+) and NPL(−), respectively, which are in good agreement with the values obtained by DLS (Figure 1b and Table S2), confirming that the interparticle forces and subsequent colloidal stability are governed by electrostatic and dispersion forces.

The possible interaction between NPLs and PFASs was assessed by ELS and DLS at various PFAS-to-NPL mass ratios. First, the oppositely charged systems, i.e., NPL­(+) together with PFAS, were studied. Note that both PFOA and PFOS are fully deprotonated under the experimental conditions applied since their p*K*_a_ values are well below the pH adjusted during sample preparation.^37^ The measured data in both the NPL­(+)-PFOA and NPL­(+)-PFOS samples are presented in Figure 2.

**Figure 2:**
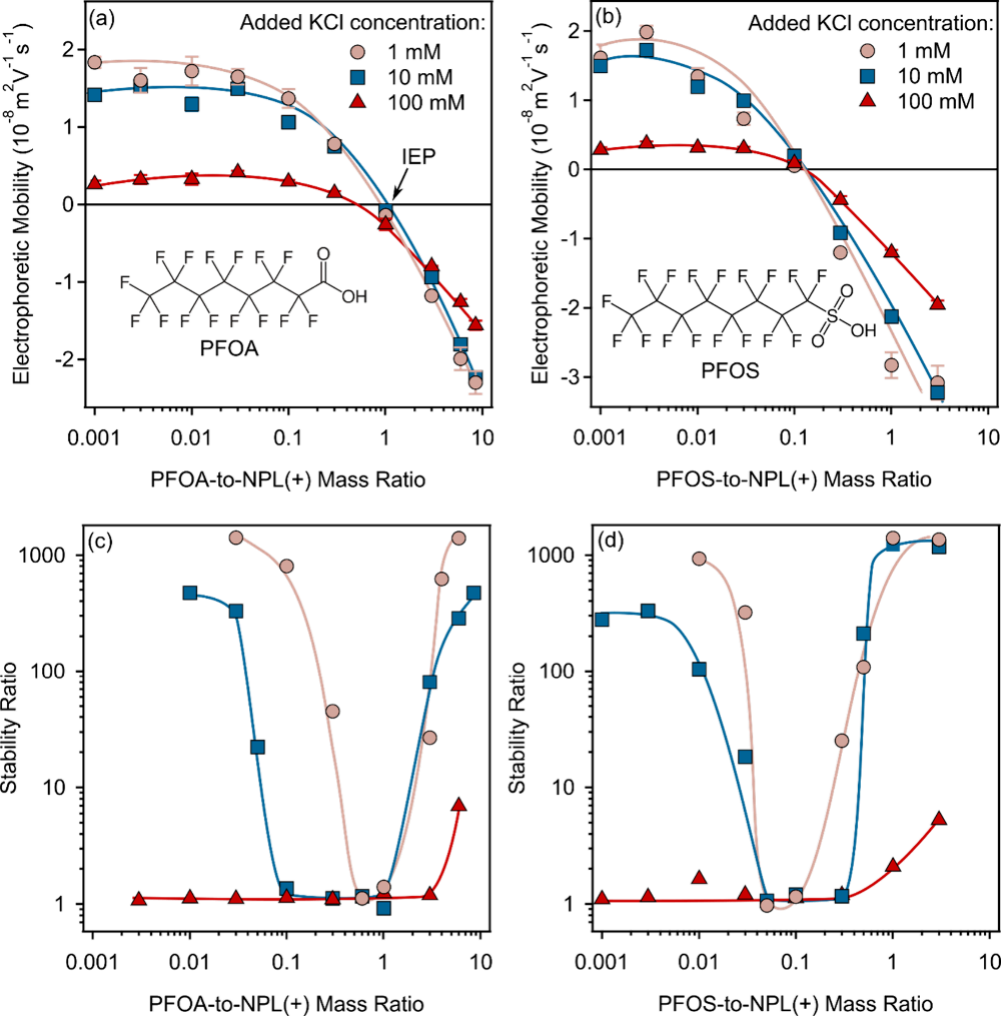
ELS electrophoretic mobility data as a function of the PFOA (a) or PFOS (b) to NPL­(+) mass ratio measured at different background electrolyte levels. Stability ratio data as a function of the mass ratio were determined in the presence of PFOA (c) and PFOS (d) at different background electrolyte levels. The NPL­(+) concentration was fixed at 10 mg/L, while the PFAS doses were varied. The solid lines serve to guide the eyes. The structures of PFOA and PFOS are in the inset of (a) and (b), respectively, and the isoelectric point (IEP) is indicated in (a).

In general, after constant values at low mass ratios, the mobilities decreased with increasing PFAS dose in both systems. The charge of the NPL­(+) particles was neutralized at PFAS concentrations corresponding to the isoelectric points (IEPs). Moreover, at high PFOA and PFOS doses, charge inversion occurred, and particles of high negative charge formed under these experimental conditions. Such trends clearly indicate the adsorption of PFASs on oppositely charged NPL­(+). Very similar charge neutralization and charge reversal were reported earlier for polystyrene particles and commercial surfactants^38^ as well as PFASs^24,26^ in oppositely charged systems, and adsorption of another PFAS was unambiguously confirmed by combining experimental and theoretical techniques.^25^ In the present case, the spontaneous attachment of PFASs to the NPL­(+) surface is mainly governed by electrostatic and hydrophobic interactions with hydrogen bonding contributing to a lesser extent.

The changes in the ionic strength affected the tendencies in the EM data similarly in both systems. At low mass ratios, i.e., at low amount of surface adsorbed PFASs, the mobilities decreased (Figure 2 and Table S3) by increasing the KCl concentration due to the screening effect^30,33^ of the salt constituents shading the EDL charge. Nevertheless, a similar screening scenario was not observed at high PFAS-to-NPL­(+) ratios, where the charge reversed particles formed. In these regimes, the EM data were insensitive to the salt concentration, which is consistent with tendencies in data reported on surfactant-latex particle interactions in the presence of background electrolytes.^39^ Such an insensitivity to the ionic strength was also obtained for the IEP values in both PFOA and PFOS samples (Figure 2a,b, respectively), as their locations were the same within the experimental error at all salt concentrations studied. Accordingly, the ionic strength affects the charge of PFAS-NPL­(+) particles at low coverage, while this influence becomes negligible at higher PFAS-to-NPL­(+) mass ratios, at which the charge is neutral or turns negative.

As discussed above, similar generic tendencies can be observed in the EM values by varying the mass ratio and salinity in both PFOA and PFOS samples. However, some system specific effects can also be identified. First, the locations of the IEP are different in the presence of PFOA and PFOS (Figure 2a,b and Table S3). The IEP is at a ten times higher mass ratio for the former one, indicating that a lower amount of PFOS is required to neutralize the NPL­(+) charge; i.e., PFOS has a higher affinity to the surface. Second, the charge reversal is more pronounced in the case of PFOS, as reflected in the EM values measured at the lowest and highest PFAS doses. Considering the same interfacial properties of the NPL­(+) in both systems, these data refer to approximately 100% and 150% extents of the charge reversal for PFOA and PFOS, respectively, compared to the bare charge of NPL­(+).

The above results indicate that PFOSs can adsorb to the surface in significantly higher amount compared to PFOAs. Both substances are classified as C8 PFASs, but they differ in their molecular masses and chemical structure of head groups. Considering these differences, one may assume that it is the higher molecular weight of PFASs that leads to the stronger adsorption in the case of PFOSs. A similar phenomenon, i.e., adsorbed amount increase with the molecular mass for compounds of similar chemical structure, was reported for other surface-active compounds such as ionic liquids,^40^ polyelectrolyte oligomers,^41^ and surfactants^42^ adsorbed on colloidal particles. Besides, the EM data imply that the PFOS has stronger affinity to the NPL­(+) surface, which can be explained by a stronger interaction between the sulfonate head groups and the surface amidine functionalities via ion correlation^43^ or hydrogen bonding.^25^ This behavior is consistent with results of previous studies demonstrating the larger adsorbed amount of PFOSs compared to PFOAs at the same analytical concentrations due to the presence of a more electronegative sulfonate headgroup that facilitates stronger electrostatic interactions with positively charged surface sites.^44,45^ However, based on the present results obtained in the oppositely charged NPL­(+)-PFAS systems, one cannot unambiguously distinguish the relative contributions of hydrogen bonding, electrostatic, and hydrophobic forces in the adsorption mechanism.

The particle aggregations in PFASs-NPL­(+) dispersions were assessed by determination of stability ratios under the same experimental conditions (concentration, pH, and ionic strength) as in the electrophoretic studies discussed above. At low salt concentrations (1 and 10 mM KCl), aggregation was slow at low PFASs-to-NPL­(+) mass ratios, was rapid in the intermediate regime, and slowed down at high doses (Figure S4a,b). Such a trend in the size data resulted in U-shape stability ratio plots at both 1 and 10 mM KCl (Figure 2c,d). The values were high (or not even measurable) at small and large mass ratios, while they were close to one in the intermediate PFAS concentration regime, which overlapped with the IEPs determined in the mobility measurements shown in Figure 2a,b. TEM images were also recorded for NPL particles treated with PFAS at doses corresponding to stability ratio values close to unity. As shown in Figure S5, compared to the bare NPLs (Figure S1), aggregated particles can be observed, confirming the results from the stability ratio measurements.

When the tendencies in the EM and stability ratio data are compared, it is evident that the aggregation is mainly governed by interparticle forces of electrostatic origin. This implies the prevalence of EDL repulsion at low and high mass ratios, where the NPL­(+)-PFAS particles possess significant charge over attractive dispersion interactions. In contrast, in the intermediate concentration regime close to the IEP, the latter forces become predominant owing to the lack of particle charge, and hence, rapid particle aggregation occurs, which destabilizes the colloids. The location of the rapid aggregation regime is shifted toward higher concentrations in the PFOA systems compared to that in PFOSs, in agreement with the shift in the IEP values discussed above. The fast aggregation rates determined at these mass ratios (Table S3) were roughly identical with the ones measured for the bare particles (Table S2) referring to the similar aggregation mechanism; i.e., the balance between EDL and van der Waals interparticle forces governed the colloidal stability. However, note that these rates are slightly lower than literature values^34,39^ due to the surfactant-free nature of the NPL­(+) particles compared to the commercial lattices used.

The situation was remarkably different at the highest ionic strength used (100 mM), but the trend was observed for both PFASs. Accordingly, NPL­(+) aggregated rapidly until high mass ratios were reached, also near the IEP, at which major deviations in the stability ratios were obtained at lower salt concentrations. At very large PFAS concentrations, moderate restabilization was indicated by the increase in the stability ratio data. Note that the ionic strength in these samples was higher than the CCC of the bare NPL­(+) (Table S2), and hence, rapid aggregation of the particles can be expected without addition of any PFASs. Considering the low (close to zero) EM data before the IEP (Figure 2), the PFAS adsorption was not able to accumulate enough charge for stabilization by EDL repulsion; thus, attractive interactions dominated, and the dispersions were destabilized. At higher PFAS loadings, the salt screening effect on the particle charge is less pronounced (see discussion with the EM data), and hence, the NPL­(+) aggregation slows due to the appearance of EDL forces. However, such a restabilization of the colloid samples is not that significant as that at lower ionic strengths because the salt screening phenomenon still plays a major role in the electrostatic features at the interface. These results highlight the importance of both the PFAS-to-NPL mass ratio and electrolyte type and concentration in the formation and aggregation of PFASs-NPL adducts, which determine the colloidal stability and, consequently, the migration and accumulation of these composite particles in the environment.

ELS and DLS experiments were performed with NPL(−) particles in the same PFAS concentration regime, ionic strength, and pH as in the case of NPL­(+). In contrast to the oppositely charged systems, no interactions between NPL(−) and the PFASs could be identified from the EM (Figure 3a,b) or hydrodynamic radii (Figure 3c,d) data.

**Figure 3:**
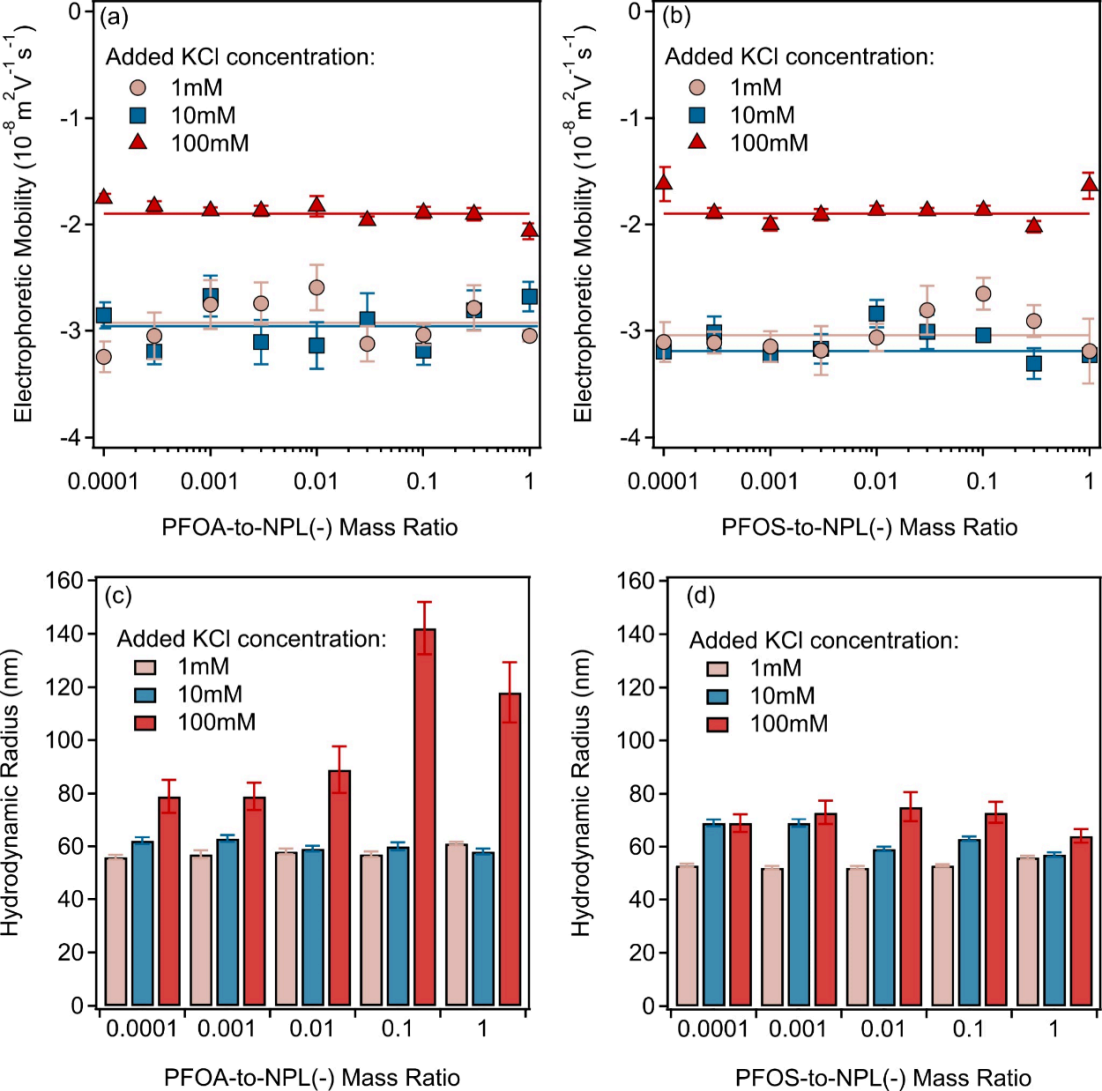
Electrophoretic mobilities of NPL(−) particles in the presence of PFOA (a) and PFOS (b) in KCl solutions. The solid lines show the average electrophoretic mobilities at the corresponding ionic strengths. Hydrodynamic radii data determined by DLS at different PFOA (c) and PFOS (d) to NPL(−) mass ratios and ionic strengths.

The ELS measurements yielded the same results with both PFOA and PFOS, and the EM data did not change significantly with the mass ratios at the respective ionic strengths. The average values (see solid lines in Figure 3a,b and data in Table S4) were the same within the experimental error as the ones determined for the bare NPL(−) particles (Figure 1a). Concerning the general trend in the hydrodynamic radii data, they increased by increasing the ionic strength due to slow particle aggregation and the presence of some aggregates, which could also be observed in the bare NPL(−) dispersion, especially at 100 mM KCl concentrations (Figure 1b). Such an aggregation was somewhat more pronounced in the presence of PFOA, but the size data indicates moderate particle aggregation and no rapid dispersion destabilization occurred under these conditions. The above results clearly show that no clear tendencies could be identified from the PFAS dose dependent EM and size data. However, one cannot unambiguously decide whether lack of change in the data is due to the absence of interaction between the PFASs and NPL(−) or whether the applied experimental techniques are just not sensitive enough to detect any PFAS-NPL(−) assemblies in the like-charged systems. Note that previous studies^46^ reported a higher extent of hydrogen bonding interactions to the adsorption process in similarly like-charged particle-PFAS systems; however, based on the present experimental results, no unambiguous evidence was obtained to confirm this hypothesis.

To conclude, the presented results demonstrate that PFAS adsorption on oppositely charged NPL­(+) induces pronounced charge regulation, leading to the charge neutralization at intermediate surface coverage and charge reversal at higher PFAS doses. The adsorption process is governed by the combined action of electrostatic attraction, hydrogen bonding, and hydrophobic interactions. Sulfonate terminated PFOS exhibits a higher surface affinity than the carboxylate head grouped PFOA, as reflected by lower IEP and more extensive charge inversion in the former case. Aggregation kinetics data revealed that colloidal stability is primarily controlled by the balance between EDL repulsion and van der Waals attraction. Stable dispersions were observed at low and high PFAS concentrations, where electrostatic repulsion dominates, whereas rapid NPL­(+) aggregation occurs near the IEP due to the suppression of electrostatic stabilization. The insensitivity of EM to ionic strength at high PFAS surface coverage of the particles further indicates a qualitative change in interfacial charging behavior compared with that of bare NPLs. In contrast, no measurable interaction was detected in like-charged systems under the experimental conditions applied, highlighting the central role of electrostatic forces in the PFAS-NPL association. Accordingly, this work establishes a quantitative link between PFAS adsorption, charge regulation, and aggregation behavior especially in NPL­(+) dispersions, while revealing system specific effects, such as distinct PFOA/PFOS affinities and an unexpected influence of ionic strength on particle charges by varying PFAS-to-NPL mass ratios, which could not be predicted by classical colloid theories. From an environmental perspective, the PFAS-induced modulation of NPL charge and aggregation identified here implies that variations in PFAS concentration and salinity can critically govern the colloidal stability, migration, and cotransport of NPLs and PFASs in aquatic environments. Besides, additional factors such as the length of the fluoroalkyl chain of PFASs and surface variations of NPLs by aging most likely affect the adsorption processes and, consequently, the colloidal stability of the samples, which can be the focus of future investigations.

## Supplementary Material



## Data Availability

All data needed to evaluate the conclusions in the paper are present in the paper and the Supporting Information.

## Notes

Experimental details; TEM images and size, PDI, EM, CCC, and charge data of NPLs and PFASs-NPLs (PDF)
