# Supplementary material for: PFAS-Induced Charge Regulation and Aggregation in Polystyrene Nanoplastic Colloids
Source: J Phys Chem Lett. 2026 Apr 30;17(19):5486–92. doi: 10.1021/acs.jpclett.6c00866 (PMC13181784; doi:10.1021/acs.jpclett.6c00866)
Supplement: Supplementary file 1 [file jz6c00866_si_001.pdf]

Supporting Information (SI)

PFAS-Induced Charge Regulation and Aggregation in Polystyrene Nanoplastic Colloids

*Tamás Péter,[†] Dóra Takács,[†] Bojana Katana,[†‡] Şeyma Miray Simav,[†] Gergő Terjéki,[†] Viktória Hornok,[†] Szilárd Sáringer,[†] Matija Tomšič,[§] and István Szilágyi[†]**

[†]MTA-SZTE Momentum Biocolloids Research Group, Department of Physical Chemistry and Materials Science, Interdisciplinary Centre of Excellence, University of Szeged, H-6720 Szeged, Hungary

[‡]Institute of Condensed Matter and Nanosciences - Bio and Soft Matter, Université catholique de Louvain, B-1348 Louvain-la-Neuve, Belgium

[§]Faculty of Chemistry and Chemical Technology, University of Ljubljana, Večna pot 113, SI-1000 Ljubljana, Slovenia

*Corresponding author. Email: szistvan@chem.u-szeged.hu

EXPERIMENTAL PART

Materials. Styrene (99%) and 2,2′-azobis(2-methylpropionamidine) (AIBA) were purchased from Acros Organics and potassium peroxodisulfate (KPS) was purchased from VWR. Heptadecafluorooctanesulfonic acid potassium salt (PFOS, $C_8F_{17}KSO_3$, ≥98.0%) and sodium perfluorooctanoic acid (PFOA, $C_8HF_{15}O_2$, ≥95.0%) were purchased from Sigma-Aldrich. Potassium chloride (KCl, 99.5%) was bought from VWR. All solutions were prepared using ultrapure water with an Adrona device and the pH was adjusted to 4 using HCl supplied by VWR. Water and salt solutions were filtered through 0.1 μm syringe filters (Millex) against dust contamination.

Synthesis of Nanoplastic Particles. The negatively (NPL(-)) and positively (NPL(+)) charged polystyrene particles were prepared by free-radical polymerization, differing only in the initiator applied. Briefly, 898 mL of ultrapure water was heated to 80 °C in a three-neck round-bottom flask equipped with a reflux condenser. The solution was bubbled with nitrogen and stirred at 500 rpm for 15 min to ensure an inert atmosphere. Subsequently, 2 g (0.2 wt%) of styrene monomer was added, and the mixture was stirred for an additional 15 min under nitrogen. In parallel, 0.2 g of initiator – KPS for the sulfate latex and AIBA for the amidine latex – was dissolved in 100 mL of nitrogen-bubbled ultrapure water and then added to the reaction mixture. The polymerization was carried out for 24 hours under continuous stirring (500 rpm) and nitrogen atmosphere. Due to the low polymer concentration (0.1-0.2 g/L), the resulting dispersion was concentrated by solvent evaporation at 50 °C until the total volume was reduced to 100 mL. The obtained dispersions were dialyzed against ultrapure water for 3 days. Dialysis continued until the conductivity of the surrounding water reached a constant value. The concentration of the final stock dispersion was 10 g/L.

Electrophoretic Light Scattering. Electrophoretic mobility (EM) was measured by a Litesizer 500 (Anton Paar) equipped with a laser source (wavelength of 658 nm) and operating at a scattering angle of 175°. The samples were prepared by mixing an appropriate amount of water, salt, PFAS, and NPL dispersion. The final volume of the samples was 2 mL and the NPL concentration was kept constant at 10 mg/L. The samples were left to equilibrate at room temperature for 2 hours prior to measurement. The electrophoretic mobility (u) of each sample was measured five times and the mean value was calculated and reported. The final value was converted to zeta potential (ζ) using the Smoluchowski equation:[1]

$$\zeta = \frac{u\eta}{\varepsilon_0 \varepsilon_r} \tag{S1}$$

where η is the viscosity of the medium, ε_0 is the dielectric permittivity of vacuum, and ε_r is the relative permittivity of water. Additionally, the surface charge density (σ) can be calculated from the ionic strength dependence of the zeta potential using the Debye-Hückel model:[2]

$$\sigma = \varepsilon \varepsilon_0 \kappa \zeta \tag{S2}$$

where κ is the inverse Debye length, representing the effect of the background electrolyte concentration on the extension of the electrical double layer.

Dynamic Light Scattering. Particle aggregation was investigated by dynamic light scattering (DLS), utilizing an ALV-NIBS/HPPS particle sizer using a 633 nm laser source. The scattered light was collected at an angle of 173°. Correlation functions were recorded for 20 seconds per measurement, with 100 runs conducted for each time-resolved experiment. The hydrodynamic radius (R_h) was determined by applying a second-order cumulant fit to the correlation function.[3] For each experiment, 2 mL dispersions were prepared following the procedure described above for the electrophoretic measurements. However, in the case of the DLS experiments, the measurements were initiated by adding the appropriate volume of the particle stock dispersion. In

the early stages of aggregation, the absolute aggregation rate constant (k) was determined from the initial change in the R_h as a function of time (t):[4, 5]

$$\frac{1}{R_{h,0}} \cdot \left(\frac{dR_h}{dt}\right)_{t \to 0} = kN_0 \left(1 - \frac{R_{h,1}}{R_{h,2}}\right) \frac{I_2(q)}{2I_1(q)} \tag{S3}$$

where $R_{h,0}$ is the initial hydrodynamic radius, N_0 is the initial number concentration of the particles, while $R_{h,1}$ and $R_{h,2}$ correspond to the hydrodynamic radius of the monomer and dimer, respectively. The contribution of the monomer (I_1) and dimer (I_2) form factors to the scattered intensity were calculated according to the Rayleigh-Debye-Gans theory.[3] The left side of the equation can be determined experimentally from the slopes of the apparent hydrodynamic radius versus time plots. The aggregation was further expressed by the stability ratio, defined as the ratio of the fast aggregation rate coefficient (k_{fast}) to the rate constant measured under the given experimental conditions:[4]

$$W = \frac{k_{fast}}{k} \tag{S4}$$

The k_{fast} was determined for both NPL systems in the presence of 1 M KCl under diffusion-controlled conditions. The destabilizing effect of a given salt was quantified based on its CCC value representing the electrolyte concentration, at which the system transitions from rapid aggregation ($W = 1$) to a stable dispersion characterized by significantly higher stability ratios ($W \gg 1$). The CCC was calculated according to the following equation:[6]

$$W = 1 + \left(\frac{CCC}{c}\right)^{-\beta} \tag{S5}$$

where c corresponds to the molar salt concentration and β was determined from the slope of the stability ratio curves within the slow aggregation regime, prior to reaching the CCC.

Transmission Electron Microscopy. TEM images were recorded with a JEOL JEM-1400Plus instrument (Japan) at 120 kV accelerating voltage. Before measurements, 10 μL sample aliquots were deposited onto carbon-coated Formvar foil 200 mesh copper grids and dried, which caused some particle aggregation during the sample preparation process.

Table S1. Composition, nomenclature and chemical properties of the PFASs.

Formula	Name (abbreviation)	Molar mass (g/mol)	Solubility in water (mg/L)[a]	pK_a[a]
$C_8HF_{15}O_2$	Perfluorooctanoic acid (PFOA)	414.07	2290	-0.5
$C_8F_{17}KSO_3$	Potassium-heptadecafluoro octanesulfonate (PFOS)	538.22	0.0032	< 1

[a]Solubility in water and pK_a values were taken from literature.[7]

Table S2. Size, polydispersity, charge and aggregation data of the NPL particles.

Particle	R_h (nm)[a]	PDI (%)[a]	R (nm)[b]	EM ($\times10^{-8} m^2V^{-1}s^{-1}$)[c]	ζ (mV)[d]	CCC (mM)[e]	σ (mCm^{-2})[f]	k_{fast} (m^3s^{-1})[g]
NPL(+)	53.2 ± 2.1	18.7 ± 1.9	40.4 ± 0.3	1.75 ± 0.24	22.6 ± 3.1	50 ± 8	4.1 ± 0.2	4.20×10^{-19}
NPL(-)	66.6 ± 2.8	20.3 ± 1.6	44.6 ± 0.2	-2.98 ± 0.36	-38.3 ± 4.6	383 ± 26	-21.1 ± 1.9	5.39×10^{-19}

[a]Hydrodynamic radius (R_h) and polydispersity index (PDI) were measured by DLS in stable dispersion at 1 mM ionic strength. [b]Determined by TEM. [c]Measured by ELS at 1 mM ionic strength. [d]Zeta potential calculated with Eq. S1. [e]Calculated by Eq. S5. [f]Particle charge density determined by fitting the salt dependent zeta potentials with Eq. S2. [g]Fast aggregation constant measured at 1 M KCl concentration in time-resolved DLS experiment using Eq. S3.

Table S3. Charge and aggregation data obtained in the dispersions of NPL(+) in the presence of PFOA and PFOS

PFAS	I mM[a]	IEP (MR)[b]	R_h (nm)[c]	PDI (%)[c]	EM ($\times10^{-8}$) ($m^2V^{-1}s^{-1}$)[c]	ζ (mV)[c]	DPC (MR)[d]	DPC (MR)[e]	k_{fast} (m^3s^{-1})[g]
PFOA	1	0.90 ± 0.30	51.1 ± 0.6	15.4 ± 13.3	-1.99 ± 0.15	-25.5± 1.9	0.98 ± 0.58	0.16	3.34×10^{-19}
	10	1.02 ± 0.94	56.3 ± 1.2	8.5 ± 3.2	-1.81 ± 0.04	-23.2 ± 0.5	0.11 ± 0.01	0.13	3.55×10^{-19}
	100	0.56 ± 0.34	144.6 ± 35.3	36.7 ± 10.0	-1.26 ± 0.04	-16.2 ± 0.5	N/A	0.00029	3.24×10^{-19}
PFOS	1	0.094 ± 0.036	50.4 ± 0.5	9.6 ± 3.6	-3.08 ± 0.25	-39.6 ± 3.2	0.045 ± 0.003	0.033	3.58×10^{-19}
	10	0.13 ± 0.08	52.5 ± 0.7	17.3 ± 4.0	-3.23 ± 0.07	-41.4 ± 0.9	0.076 ± 0.023	0.042	3.00×10^{-19}
	100	0.14 ± 0.02	131.5 ± 77.9	67.9 ± 41.8	-1.96 ± 0.06	-25.1 ± 0.8	N/A	0.00018	3.07×10^{-19}

[a]Ionic strength (I) Adjusted by KCl. [b]Isoelectric point expressed in mass ratio (MR) and measured by ELS. [c]Obtained at 6 and 3 PFAS-to-NPL(+) mass ratios for PFOA and PFOS, respectively. Destabilization PFAS concentration measured by DLS[d] and calculated[e] by Ref. Galli et. al. [f]Fast aggregation rate constant determined at 1 and 0.1 mass ratios for PFOA and PFOS, respectively, in time-resolved DLS experiment using Eq. S3 for calculation. N/A = not applicable.

Table S4. Charge and size data obtained in the dispersions of NPL(-) in the presence of PFOA and PFOS

PFAS	I mM[a]	R_h (nm)[b]	PDI (%)[b]	EM (×10^{-8}) ($m^2V^{-1}s^{-1}$)[c]	ζ (mV)[c]
PFOA	1	61.3 ± 0.7	6.1 ± 3.9	-2.92 ± 0.20	-37.5 ± 2.5
	10	57.8 ± 1.0	6.4 ± 3.7	-2.95 ± 0.19	-37.9 ± 2.4
	100	117.9 ± 11.2	27.9 ± 7.4	-1.90 ± 0.08	-24.3 ± 1.1
PFOS	1	55.8 ± 0.6	12.2 ± 4.9	-3.04 ± 0.18	-39.2 ± 2.6
	10	56.9 ± 0.9	9.5 ± 12.9	-3.19 ± 0.26	-40.9 ± 3.4
	100	63.8 ± 2.5	15.9 ± 6.5	-1.90 ± 0.19	-24.4 ± 2.4

[a]Ionic strength (I) adjusted by KCl. [b]Measured by DLS at 1 PFAS-to-NPL(-) mass ratios. [c]ELS average value taken from the data obtained in the entire PFAS concentration range applied.

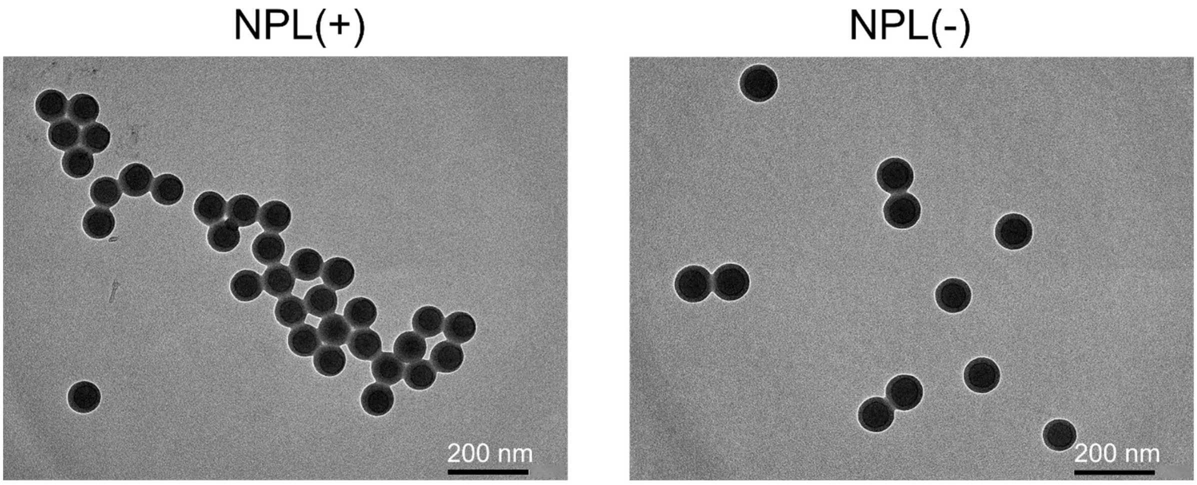

Figure S1. TEM images of NPL(+) (left) and NPL(-) (right) taken in dried state after solvent evaporation.

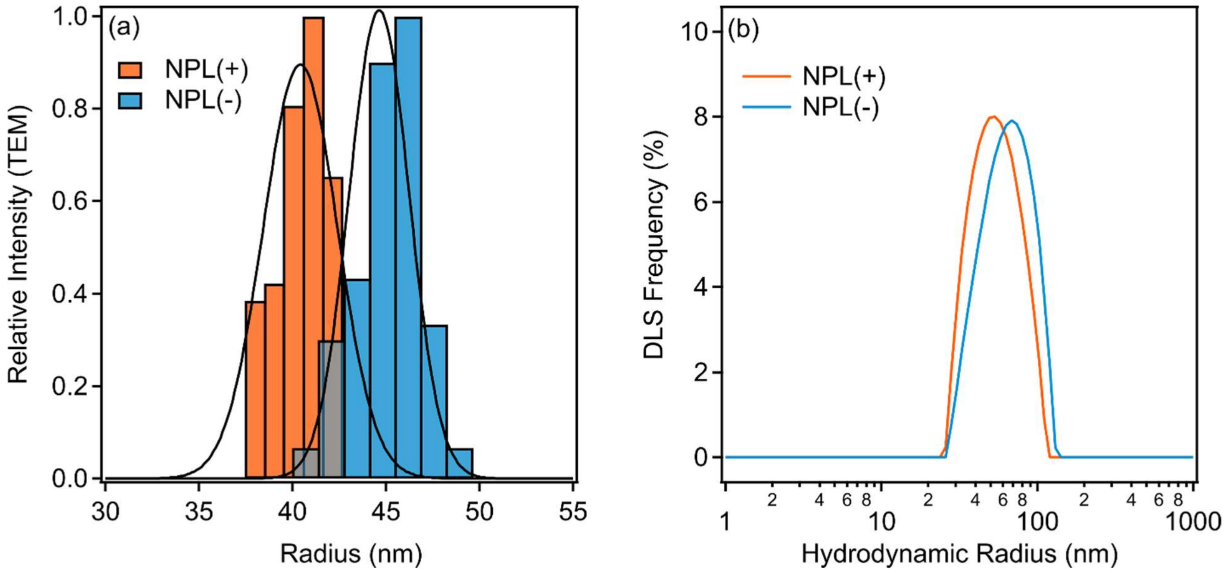

Figure S2. Size distributions of NPLs determined by TEM (a) and DLS (b, measured at pH 4 and ionic strength of 1 mM).

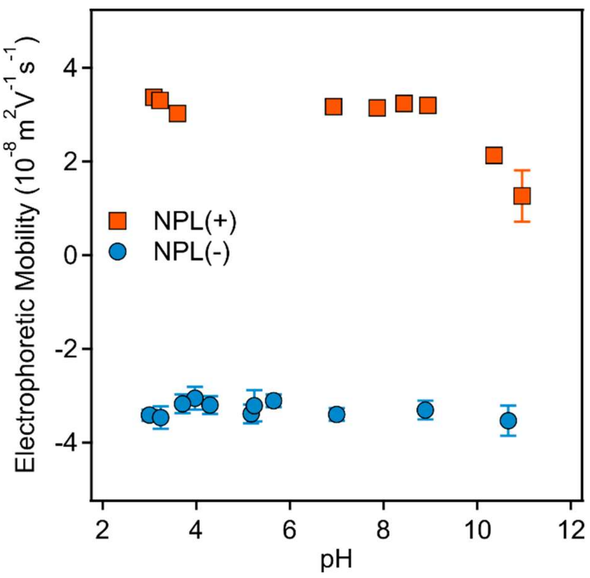

Figure S3. The pH dependent electrophoretic mobility data determined for the NPLs at 1 mM ionic strength.

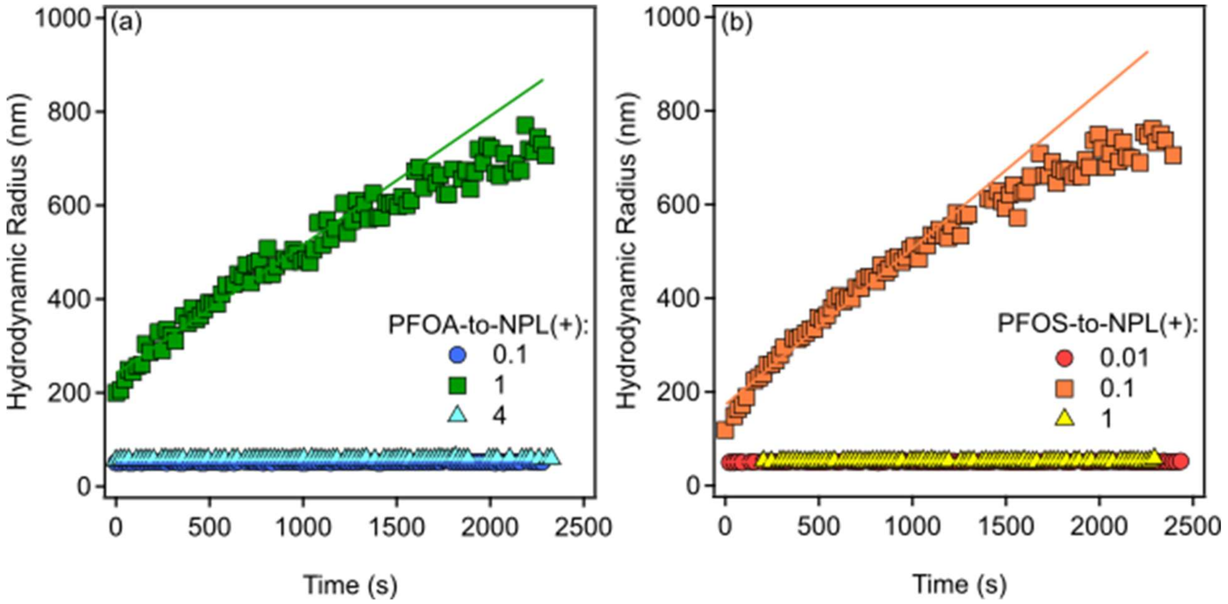

Figure S4. DLS hydrodynamic radii versus time data for PFOA (a) and PFOS (b) at different PFAS-to-NPL(+) mass ratios and 1 mM ionic strength.

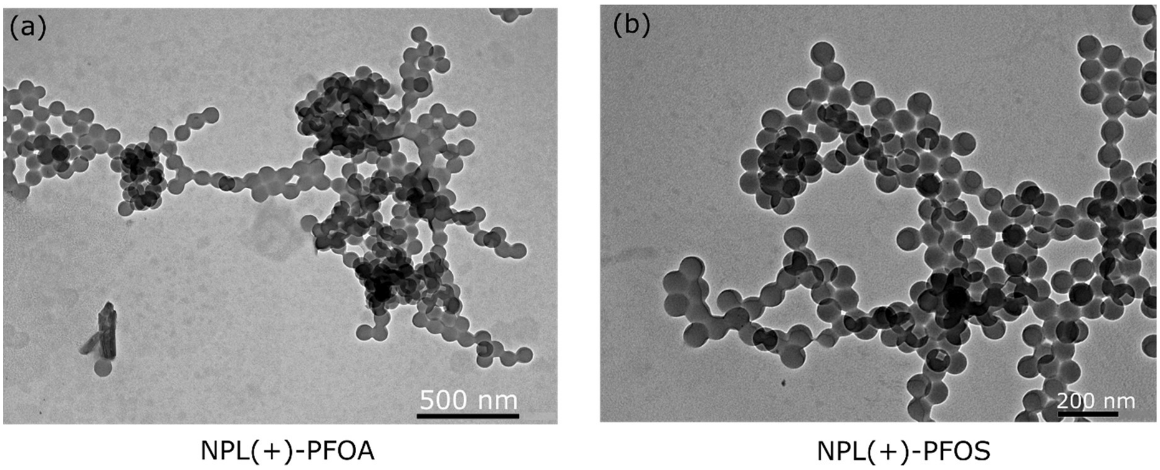

Figure S5. TEM images taken in the NPL(+)-PFOA (a), NPL(+)-PFOS (b) dispersions at 1/0.1 PFOA/PFOS-to-NPL mass ratios.

REFERENCES

(1) Delgado, A. V.; Gonzalez-Caballero, F.; Hunter, R. J.; Koopal, L. K.; Lyklema, J. Measurement and interpretation of electrokinetic phenomena. *J. Colloid Interface Sci.* **2007**, *309*, 194-224.

(2) Trefalt, G.; Szilagyi, I.; Tellez, G.; Borkovec, M. Colloidal stability in asymmetric electrolytes: Modifications of the Schulze-Hardy rule. *Langmuir* **2017**, *33*, 1695-1704.

(3) Holthoff, H.; Egelhaaf, S. U.; Borkovec, M.; Schurtenberger, P.; Sticher, H. Coagulation rate measurements of colloidal particles by simultaneous static and dynamic light scattering. *Langmuir* **1996**, *12*, 5541-5549.

(4) Trefalt, G.; Szilagyi, I.; Oncsik, T.; Sadeghpour, A.; Borkovec, M. Probing colloidal particle aggregation by light scattering. *Chimia* **2013**, *67*, 772-776.

(5) Takács, D.; Tomšič, M.; Szilagyi, I. Effect of water and salt on the colloidal stability of latex particles in ionic liquid solutions. *Colloid Interfac.* **2022**, *6*, 2.

(6) Grolimund, D.; Elimelech, M.; Borkovec, M. Aggregation and deposition kinetics of mobile colloidal particles in natural porous media. *Colloid Surf. A* **2001**, *191*, 179-188.

(7) Gagliano, E.; Sgroi, M.; Falciglia, P. P.; Vagliasindi, F. G. A.; Roccaro, P. Removal of poly- and perfluoroalkyl substances (PFAS) from water by adsorption: Role of PFAS chain length, effect of organic matter and challenges in adsorbent regeneration. *Water Res.* **2020**, *171*, 115381.
